# Cultural theory and political philosophy: Why cognitive biases toward ambiguous risk explain both beliefs about nature's resilience and political preferences regarding the organization of society

**DOI:** 10.1111/risa.17668

**Published:** 2024-10-29

**Authors:** Marc D. Davidson

**Affiliations:** ^1^ Department of Ethics and Political Philosophy Radboud University Nijmegen The Netherlands

**Keywords:** ambiguous risk, cognitive biases, cultural theory of risk, planetary boundaries, political philosophy

## Abstract

Many studies have observed a correlation between beliefs regarding nature's resilience and (political) preferences regarding the organization of society. Liberal‐egalitarians, for example, generally believe nature to be much more fragile than libertarians, who believe nature to be much more resilient. Cultural theory explains this correlation by the idea that people are only able to see those risks that fit their preferred organization of society. This article offers an alternative, second explanation for the observed correlation: Both beliefs regarding nature's resilience and political preferences can be explained by the same cognitive biases toward ambiguous risk, that is, dispositions determining our expectations regarding the possible state of affairs resulting from our acts and their probabilities. This has consequences for political philosophy and the psychology of risk. In particular, there is a knowledge gap in psychology regarding the cognitive biases underlying the belief that despite ambiguity, experts can determine safe limits for human impacts on the environment.

## 1 INTRODUCTION

Knowledge is ever expanding, but a point always remains where the eye of science as yet cannot see. In this epistemic mist, we do not know all possible states of affairs that may result due to our acts let alone their probability. Climate change is a case in point. Although the Intergovernmental Panel on Climate Change concludes that “it is unequivocal that human influence has warmed the atmosphere, ocean, and land” (IPCC, 2021: 4), the reasons for concern with further global warming are ambiguous. The IPCC offers neither a full overview of all possible ecological and social impacts of climate change nor quantified probabilities that these impacts will materialize. The epistemic mist is thickest regarding existential risk to the human species and the expectations that technological solutions such as geoengineering will save us.

According to cultural theory (CT), people facing such ambiguous risks perceive the world through a “filter” influencing both the way issues are defined as well as preferences as to how they should be handled (see, e.g., Adams, 1995; Douglas & Wildavsky, 1982; Johnson & Swedlow, 2021; Schwarz & Thompson, 1990; Thompson *et al.*, 1990). Moreover, CT states that beliefs regarding nature's resilience and values regarding the organization of society and thus political preferences are correlated. Liberal‐egalitarians, for example, generally believe nature to be much more fragile than libertarians, who believe nature to be much more resilient (see also Buttel & Flinn, 1978; Cruz, 2017; Ellis & Thompson, 1997). CT explains this correlation by the idea that people's values regarding the organization of society guide and therefore precede their perception of risk (Douglas, 1985: 60; Douglas & Wildavsky, 1982; Kahan & Braman, 2006; Thompson *et al.*, 1990). Libertarians, for example, would reject the existence of a climate crisis because its acknowledgment would simultaneously require the acknowledgment of the need for governmental intervention, which conflicts with the libertarian preferred organization of society. CT is not restricted to environmental risks but applies to any ambiguous situation, such as financial risks (Linsley & Shrives, 2009), new technologies (Tansey & Rayner, 2009), or the COVID‐19 pandemic (Dimitrijevska‐Markoski & Nukpezah, 2023).

The objective of this article is to propose an alternative, second explanation for the observed correlation between beliefs regarding nature's resilience and values regarding the organization of society: Both can be explained by the same cognitive biases toward ambiguous risk, that is, dispositions determining our expectations regarding the possible state of affairs resulting from our acts and their probabilities.^1^ It should be emphasized that this article offers an explorative argument and intends to raise discussion and invite further empirical and theoretical research by establishing a stronger link between CT on the one hand and political philosophy and psychology on the other. Two hypotheses in particular are forwarded. First, that cognitive biases toward ambiguous risk inevitably influence theories and conceptions of (distributive) justice, which is particularly relevant for political philosophy. Second, that underexplored cognitive biases underlie the belief in “planetary boundaries,” which is particularly relevant for psychology.

The setup of this article is as follows. Section 2 first describes CT in more detail. Section 3 aims to make plausible that four sets of cognitive biases toward ambiguous risk underlie the four myths of nature's resilience distinguished in CT. Section 4 aims to make plausible that these same four sets of cognitive biases inevitably influence political preferences regarding the organization of society and conceptions of justice and thus link to the four myths of human nature distinguished in CT. Section 5 concludes and discusses paths for further research.

## 2 THE CULTURAL THEORY OF RISK

CT is composed of three main claims. The first claim is that there are four basic archetypical beliefs about the world or myths of nature. The second claim is that there are also four basic archetypical social relationships or ways of life called myths of human nature. The third claim is that these two sets of archetypes correlate and that this correlation can be explained in terms of the contribution that risk perceptions have for maintaining a particular way of life. These three claims are discussed in more detail below.

### 2.1 Myths of nature

The first claim of CT is that people differ in their *beliefs* about what is “out there” in the epistemic mist and that these beliefs often match one of four archetypes called myths of nature (Holling, 1978, 1986; Schwarz & Thompson, 1990). The first myth is that nature is ephemeral or fragile: if we disturb nature, it may get out of balance leading to catastrophic results such as a runaway greenhouse effect. The accompanying approach to risk is precautionary: Do not move in the epistemic mist unless you are certain that it is safe. The rationale behind the precautionary approach is that once dangers materialize solutions are not easily found. Technology will not save us and if it does for a particular problem, it will create problems elsewhere (Tenner, 1997; Coad *et al.*, 2021). Think of nuclear energy, which hardly produces carbon dioxide but cannot be developed in time on the required scale and will introduce other risks, such as accidents, nuclear waste, and the proliferation of nuclear arms.

The second myth is that nature is benign and stable: if we disturb nature, it will find its initial state again. The accompanying approach to risk is trial and error: Simply move inside the epistemic mist, because if unexpected dangers do materialize one can simply retrace one's steps or find solutions. As history has shown, human ingenuity is limitless (Lomborg, 2003; Pinker, 2018; Rand, 1971; Ridley, 2010; Rosling, 2019; Simon, 1996). Malthus (1798) and Ehrlich (1968), who warned of overpopulation, famine, and collapse, did not foresee the Green Revolution (Evenson & Gollin, 2003), for example. Per capita food production is now higher than at the time of either Malthus or Ehrlich (Roser *et al.*, 2023).^2^

The third myth is of nature as perverse/tolerant. At first sight, this myth may appear as a middle ground between the previous two: the idea that there is a safe zone. After a disturbance within certain limits, nature will indeed find its initial state again, but as we go beyond these safe limits, we will still head toward a catastrophe. The peculiarity about this view, however, is its denial of the epistemic mist: If we press experts, in spite of uncertainty and indeterminacy, they are able to quantify this ecological utilization space (Opschoor, 1987; Siebert, 1982), “safe operating space for humanity,” or “planetary boundaries” (Biermann & Kim, 2020; Rockström *et al.*, 2009). The same holds for finding solutions when we move in the epistemic mist: Governments can manage and govern technological transitions (Biermann, 2007; Meadowcroft, 2009).

The final myth is of nature as capricious. Basically, we do not have a choice about moving in the epistemic mist. Neither nature nor technology can be managed. History will have its course whatever we do and the same holds for nature. It is a lottery, one does not know what to expect, and one cannot learn from experience (Mamadouh, 1999).

Of course, in a public debate, we may encounter rhetoric: Environmental activists may exaggerate scientific certainty about environmental risks, whereas the fossil‐fuels industry may exaggerate the epistemic mist where science already for long has a clear view. Nevertheless, stripped from such rhetoric fundamentally different beliefs regarding nature's resilience remain. The essence of CT is that there is no “truth” or “untruth” in any of these archetypes, as these beliefs by definition hold in the epistemic mist, that is, where the eye of science cannot yet reach.

### 2.2 Myths of human nature

The second claim of CT is that people differ in their social relationships or ways of life and these also often match four archetypes, called myths of human nature. The four archetypes are based upon the grid‐group typology first developed by Douglas (1978, 1982) and further elaborated by Schwarz and Thompson (1990) and Thompson *et al.* (1990). In this typology, grid “denotes the degree to which an individual's life is circumscribed by externally imposed prescriptions,” and group “refers to the extent to which an individual is incorporated in bounded social units” (Thompson *et al.*, 1990:5). These two dimensions of grid and group lead to four ways of life or archetypes that differ in their fundamental values, their interpretation of fairness, their allergies, and where to look for the fundamental causes when things go wrong and thus also for solutions. There is thus a strong link between the myths of human nature and political preferences. As Swedlow *et al.* (2020: 2334) note, “Each of these four patterns of social and political relations is hypothesized to justify and in turn be justified by specific political values and beliefs.”

The first archetype with low grid and high group orientation is that of the egalitarian, who favors the leveling of the social pyramid. Fairness for the egalitarian implies that at the end of the day, people end up with equal benefits of cooperation. The egalitarian fights against the exploitation of some groups by others and is thus allergic to exploitation of the weaker by those in power and the less wealthy by the more wealthy. If things go wrong, blame is put on the “system,” because systemic flaws made the occurrence of the problem inevitable.

The second archetype with low grid and low group orientation is that of the individualist who tends to prefer a laissez‐faire lifestyle, favoring few controls beyond those required to establish and maintain property rights and control criminal behavior. For the individualist, the autonomy of the individual is the highest political and social good. Fairness consists of equality of opportunity. The individualist is allergic to parasitism: The misuse of social services like unemployment benefits, for example, by those who do not need them. If things go wrong, blame is put on (accidental) personal failure, and there is no need to look any further.

The third archetype with high grid and high group orientation is that of the hierarchist who considers the collective wellbeing of society more important than individual rights. Fairness consists of equality of consideration under the established rules that have proven to promote the collective interest. The hierarchist is allergic to deviating from these established rules. If things go wrong, blame is put on individuals who failed to follow the established rules or on the lack of those rules.

The last archetype with high grid and low group orientation is that of the fatalist. Fatalists are generally apathetic about how society is structured, believing it to be inevitable that powerful groups will exploit weaker groups. Fairness is not to be found on this earth. If things go wrong, blame is put on fate (bad luck).

### 2.3 Correlation and explanation

The third claim of CT is that the two previously mentioned groups of archetypes *correlate* (Schwarz & Thompson, 1990). It is generally the egalitarian who believes nature is fragile and adopts the precautionary principle, the individualist who believes nature is robust and immune to trial and error, the hierarchist who believes scientists can quantify a safe operation space, and the fatalist who believes it all does not matter. Empirical evidence for CT has been offered by various studies (see, e.g., Dake, 1991; Ellis & Thompson, 1997; Hartmann, 2012; Johnson & Swedlow, 2020; Kahan *et al.*, 2012, 2006; Langford *et al.*, 2000; Peters & Slovic, 1996; Poortinga *et al.*, 2002; Sjöberg, 1997; Verweij & Thompson, 2006), although the evidence has also been contested (see, e.g., Boholm, 1996; Marris *et al.*, 1998; Oltedal *et al.*, 2004; Sjöberg, 1998, 2002).

Apart from this empirical evidence from studies among the general public, the correlation between risk perception and values can easily be discerned in political, public, and academic debate. Political philosophers, for example, who worry about the state of the environment, such as Caney (2010), and Gardiner (2011), Shue (1999) often follow the liberal‐egalitarian footsteps of Beitz (1975), and Dworkin (1981), Rawls (1971). Libertarians by contrast are generally silent on environmental matters (Wissenburg, 2019) or downplay perceived environmental problems such as Narveson (1998: 98). Climate sceptics often consider (supranational) governments imposing climate regulation the first brick of *The Road to Serfdom* (Hayek, 1944); climate “alarmists” generally stress how particularly the least‐advantaged in global society are most vulnerable and how climate taxes could help redistribute wealth. In the political spectrum, environmental concerns have a higher priority for the political left than the political right, and there are few votes for politicians and political parties that combine libertarian views with a deep concern about the environmental state of our planet or, on the contrary, combine egalitarian views with disinterest in environmental concerns, although the latter is more common than the first.

The archetypes correlate, according to CT, because (preferred) social relationships precede and determine risk perceptions (Dake, 1992; Johnson & Swedlow, 2021; Kahan & Braman, 2006; Thompson *et al.*, 1990).^3^ In the words of Douglas (1985, p. 60): “moral concern guides not just response to the risk but the basic faculty of [risk] perception.” According to Douglas and Wildavsky (1982), various structures of social organization endow individuals with perceptions that reinforce those very structures, in competition against alternative structures of organization. Risk perceptions are thus explained in terms of the contribution that those perceptions have for maintaining a particular way of life in the grid/group dimensions. Individualists, for example, would proclaim a cornucopian view of nature because an environment at danger would require government regulation that is at odds with the individualist's preferred laisser‐faire economy. It is often assumed that the denial of man‐made climate change is fed by the fear that climate mitigation would require big government, or even worse: regulation imposed by an international body (see e.g. Oreskes & Conway, 2011). The hierarchist's idea that there is a safe zone that can be determined by experts would be functional for the hierarchist's preferred top–down management of hazards. Egalitarians might view environmental dangers as an opportunity to address fundamental injustice in society and therefore subconsciously have reason to enlarge these dangers. It is from this point of view that CT derives its name: Risk perception would be culturally determined.

CT does not explain how people come to adopt a particular cultural perspective or political preference (Breakwell, 2014: 81). It can also be argued, however, that both beliefs/perceptions regarding nature's resilience and political preferences regarding the organization of society are driven by the same cognitive biases toward ambiguous risk. These expectations and attitudes partly depend upon the characteristics of the risks involved (e.g., Fischhoff *et al.*, 1978; Sjöberg, 1996; Slovic, 1987) but are also person‐specific subjective or predisposed opinions that may emanate from specific heuristics. In the next section, I start with describing the cognitive biases underlying the myths of nature as distinguished in CT, that is, the four more general beliefs about what hides in the epistemic mist (i.e., how we assess the probabilities of various possibilities), how we value what we believe to be hiding, and what will result due to our choices. Section 4 will then argue that these cognitive biases toward ambiguous risk inevitably influence political preferences regarding the organization of society and conceptions of justice.

## 3 COGNITIVE BIASES AND BELIEFS REGARDING NATURE'S RESILIENCE

The objective of this section is to make plausible that four sets of cognitive biases toward ambiguous risk explain the four myths of nature's resilience as distinguished in CT. Please note that only general cognitive biases are discussed, such as pessimism or the illusion of control, that is, biases that do not depend upon the specific circumstances, and that other cognitive biases that do depend on specific circumstances are considered less relevant, such as availability bias and anchoring (Tversky & Kahneman, 1974). Moreover, note that cognitive biases are sometimes defined in the literature as systematic discrepancies between one's judgment and the “correct” answer, but that in the case of ambiguous risk, there is no correct answer. Perceiving the glass to be half full, for example, reflects a cognitive bias but not an error of judgment.

### 3.1 Nature as fragile

The myth of nature as ephemeral or fragile is best explained by the cognitive biases of pessimism, ambiguity aversion, and risk aversion. In the case of ambiguous risk, pessimism is the general tendency to see more negative possibilities (bad outcomes) than positive possibilities and, moreover, to attach higher probabilities that these negative possibilities will materialize (see e.g. Lopes, 1987). The same pessimism applies to the likelihood that if problems occur, solutions will be found and to the new unforeseen, negative side‐effects that these solutions will introduce. The pessimist will therefore take a precautionary approach by trying to avoid moving in the epistemic mist or move slowly so as not to increase further ambiguity. Ambiguity aversion is therefore related to pessimism: the tendency of individuals to prefer certain risks over ambiguous risks (Elssberg, 1961). People may wish to avoid ambiguous situations because they feel uncertain or uncomfortable with the lack of information: “The devil you know is better than the devil you do not know. ” People with a pessimistic cognitive bias will also often show loss‐ or risk‐aversion, that is, attaching a higher weight to the worst outcomes than to the best outcomes (Arrow, 1963; Pratt, 1964). An example of risk‐averse behavior is choosing a certain 100 Euro instead of tossing a coin with an equal probability of winning 300 Euro or nothing. Risk‐aversion may be rationally based on the diminishing marginal utility of goods (Bernoulli, 1738; Vickrey, 1945), but also on irrational pessimism that despite certain equal probabilities, such as in the case of tossing a coin, a negative outcome is more likely. Note that pessimism and risk‐aversion are distinct: In theory, one can be pessimistic about possibilities and their probabilities but still be risk‐seeking.

### 3.2 Nature as benign

The myth of nature as benign is best explained by the cognitive biases of *optimism* (Weinstein, 1980) and being risk‐seeking. Optimism refers to the opposite of the previously described pessimism, that is, the general tendency to expect good outcomes in the case of ambiguous risk. The optimist expects no dangers looming in the mist (the probability of problems is assessed as low) and believes that if we nevertheless encounter problems, we will find solutions. The optimist moreover sees a potential to be exploited in the mist: if we are risk‐seeking, we attach a higher weight to the best outcomes than to the worst outcomes. We therefore move in the mist with confidence and proceed on the basis of trial‐and‐error.

### 3.3 Nature as perverse/tolerant

The myth of nature as perverse/tolerant is best explained by risk neutrality in combination with the cognitive bias of overconfidence, a failure to know the limits of one's knowledge (Oskamp, 1965). In particular, this myth expresses overconfidence in the ability of experts to optimize our response on the basis of quantitative data in defiance of ambiguity. The overconfidence bias is based on mental heuristics to reduce the complexity of judgment (Kahneman *et al.*, 1982). So the myth of nature as perverse/tolerant does not only refer to the belief in the existence of ecological tipping points that can be quantified by natural scientists, but also to the belief that economists can quantify optimal levels of environmental impact on the basis of cost‐benefit analysis.^4^ Climate change is a case in point: Although it is certain that climate risks increase with rising global temperatures and quite certain that these risks increase nonlinearly, that is, faster at higher temperatures, the IPCC does not indicate a tipping point where temperatures change from “safe” to “unsafe.” Nevertheless, two degrees of temperature increase above preindustrial levels is used in many publications as such a tipping point (see, e.g., Rockström *et al.*, 2009; see, e.g., Knutti *et al.*, 2016 on criticism of the two degrees target).^5^ Moreover, natural scientists and economists are not only deemed capable of optimizing our response, but experts can also manage finding solutions.

### 3.4 Nature as a lottery

The myth of nature as a lottery (“capricious” in CT) can be explained by both the cognitive bias of a low internal locus of control, that is, a low expectancy pertaining to the connection between personal characteristics and/or actions and experienced outcomes (Langer, 1975; Rotter, 1954 and 1966), and by the philosophical belief of fatalism that the occurrence of problems does not depend upon our decisions and thus are inevitable. Fatalism differs from pessimism in the fact that the pessimist believes dangers can be avoided or reduced by precaution, whereas the fatalist would deny that such precaution matters. Although the fatalist might be agnostic about the nature of fate, in CT she believes fate to be spiteful, that is, the fatalist in CT is also cynical.

## 4 COGNITIVE BIAS AND CONCEPTIONS OF JUSTICE

The objective of this section is to argue that cognitive biases toward ambiguous risk inevitably influence political preferences regarding the organization of society and conceptions of justice, not only as a psychological matter of fact but also in normative political philosophy. As will be shown, the cognitive biases toward ambiguous risk discussed in the previous section are strongly related to the political theories of liberal egalitarianism, libertarianism, and utilitarianism, which in turn are strongly related to the values held by respectively the egalitarian, individualist, and hierarchist in CT.^6^

Most political philosophers agree that principles of justice should not be tailor‐made to the personal benefits we would reap under such principles given the specific situation in which we accidently find ourselves (see, e.g., Kymlicka, 2002). To think about justice and the organization of society, we have to ask ourselves how we would organize society if we did not know who we would be in that society and which position we would occupy.^7^ We have to put ourselves in other people's shoes as well. This idea has a long pedigree. It has been proposed, for example, by Vickrey (1945) and Harsanyi (1953) and more in general expresses the idea of universalizability, that is, that moral judgments require taking an impartial perspective, which has been defended by amongst others David Hume (1740), Adam Smith (1759) and Immanuel Kant (1785). The thought experiment has become most well‐known, however, through the work of the political philosopher John Rawls. In A Theory of Justice (1971), Rawls asks us to imagine a hypothetical situation or “original position” in which people have to select principles that will determine the basic structure of the society they will live in behind a “veil of ignorance” (1971: 181):

no one knows his place in society, his class position or social status; nor does he know his fortune in the distribution of natural assets and abilities, his intelligence and strength, and the like. Nor, again, does anyone know his conception of the good, the particulars of his rational plan of life ….

The veil of ignorance guarantees that the chosen principles of justice will be fair, that is, that people are treated as moral equals. Not only do political philosophers normatively require such an impartial perspective on justice, it also corresponds to common moral intuitions about fairness, reciprocity, and justice (Trivers, 1971).

Choosing principles of justice behind a “veil of ignorance” is preeminently a matter of ambiguous risk. After all, we have neither a complete view of all the positions that we can occupy in society nor knowledge of the probabilities of these positions. We may have a moral duty to educate ourselves as much as possible about the lives of others in the world, but our assessment of alternative circumstances is unavoidably epistemologically limited. Consequently, the choice of principles can never be a mere matter of rational choice and optimization. According to Savage (1954), the father of modern Bayesian decision theory, such optimization would only be possible in “small worlds” where all alternatives, consequences, and probabilities are known with certainty, but not in “large worlds” where not all is known and surprises can happen. In the case of such bounded rationality, we can better satisfice on the basis of heuristics instead of optimization (Gigerenzer, 2010). Choosing behind the veil of ignorance clearly belongs to the realm of large worlds and the chosen principles will therefore inevitably reflect our individual cognitive biases, such as optimism and pessimism.

The inevitability of cognitive bias is overlooked or ignored, however, in most literature discussing decision making behind the veil of ignorance (see, e.g., Angner, 2004; Buchak, 2017; De Coninck & Van De Putte, 2023; Gaus & Thrasher, 2015; Gustafsson, 2018; Harsanyi, 1953, 1975; Peres, 2021; Rawls, 1971).^8^ Although Rawls himself understood and emphasized that choosing behind the veil of ignorance is a matter of ambiguity (Rawls, 1971: 154), he unjustly believed that we could detach ourselves from cognitive biases. Rawls thus added the requirement that behind the veil of ignorance one does not know “even the special features of his psychology such as his aversion to risk or liability to optimism or pessimism” (1971: 181; see also Lambert & Weale, 1981). This, however, stretches the human imagination too far (Levin, 1978: 152). The essence of cognitive biases toward ambiguous risk is precisely that it is impossible to abstract from them, as is also the central thought in CT. We cannot remove the filter through which we look at the world, as there does not exist a “neutral” risk perception in the case of ambiguity. Under the circumstance of true uncertainty and indeterminacy, there is no “rational” choice as if one's risk filter is a matter of uncertainty itself for which we can insure ourselves or a preference we can imagine not to have. The question, after all, is not whether we can imagine what it is like to organize society with different cognitive biases—we can, as will be shown below—but whether we can imagine how without cognitive biases we would organize society differently if we could end up with different cognitive biases. The latter is meaningless. Behind the veil of ignorance, we will inevitably choose on the basis of our personal risk attitude or cognitive bias. Cognitive biases toward risks such as optimism or pessimism precede any handling of ambiguous risk.

Cognitive biases do not only play an important role in the thought experiment of the veil of ignorance, but also if we start moral reasoning with theoretical principles instead. According to Nozick (1974), for example, society should be organized according to libertarian principles so as to reflect the deontological principle that individuals are ends and not merely means. According to Bentham (1781), society should be organized according to the utilitarian principle that overall wellbeing, utility, or happiness should be maximized. There is no inherent truth in theoretical principles, however, independent from consistency with our considered moral judgments or intuitions in specific cases (Knight, 2023; Rawls, 1971). If our moral intuitions regarding a specific situation or case do not match the recommendations of the moral or political theory we hold to be correct, we either have to reject the theory and look for an alternative or have a second look at our intuitions whether they stand the test of further judgment. In either case, all we can strive for is coherence between theory and intuitions, that is, reflective equilibrium. For Rawls (1971), the conflict between the theory of utilitarianism and his moral intuitions regarding its practical consequences was the reason to reject utilitarianism. As these moral intuitions are related to our tendencies to put ourselves in other people's shoes, they are influenced by the same cognitive biases that influence our decisions behind the veil of ignorance.^9^

In the following paragraphs, it will be argued that if we move behind the veil of ignorance with the four sets of cognitive biases toward ambiguous risk as distilled in Section 3, indeed the standard positions in political debate and philosophy result. About the fatalistic position, we can be short: If we do not believe that our decisions determine the outcomes, that is, if we lack control, we will also be apathic about the organization of society.

### 4.1 Pessimism, ambiguity aversion, risk aversion, and liberal‐egalitarianism

Rawls’ own work offers a clear example how the cognitive biases of pessimism, ambiguity aversion, and risk aversion result in liberal‐egalitarian principles of justice. Rawls assumes that behind the veil of ignorance, people will play safe: If we do not know who we are in society and we thus can occupy the worst position, then we will organize society in such a manner that the worst position is made as good as possible. To be more precise, Rawls formulates two principles of justice (1971: 302):

FIRST PRINCIPLE Each person is to have an equal right to the most extensive total system of equal basic liberties compatible with a similar system of liberty for all.

SECOND PRINCIPLE Social and economic inequalities are to be arranged so that they are both: (a) to the greatest benefit of the least advantaged, … and (b) attached to offices and positions open to all under conditions of fair equality of opportunity.

Although Rawls states that the first principle has lexical priority over the second principle, the basic liberty to the fruits of one's own labor and personal property is loosened so as to be able to redistribute wealth to the least‐advantaged in society. The idea that social and economic inequalities are to be arranged so that they are to the greatest benefit of the least advantaged is called the difference principle. The first principle distinguishes Rawls’ liberal egalitarianism from utilitarianism, whereas the difference principle in particular distinguishes it from libertarianism.^10^

Rawls himself argues that his choices behind the veil of ignorance and the precautionary approach on which they are based are not pessimistic, but rational instead. He offers three arguments (Rawls, 1971: 154): (I) “The situation is one in which a knowledge of likelihoods is impossible, or at best extremely insecure.” (2) “The person choosing has a conception of the good such that he cares very little, if anything, for what he might gain above the minimum stipend that he can, in fact, be sure of by following the maximin rule.” (3) “The rejected alternatives have outcomes that one can hardly accept.” The second and third arguments clearly express pessimism, however, by respectively downplaying positive outcomes and overemphasizing negative outcomes. It is difficult to imagine that people will “care little, if anything” about the options available to the Zuckerberg's and the Musk's in a world without any wealth redistribution in comparison to a world based upon the egalitarian difference principle. This is particularly true given the fact that behind the veil of ignorance, we are assumed to have no knowledge of our conception of the good. Likewise, it can be doubted whether it is possible to reject an outcome as “unacceptable” without any knowledge of its probability and what might be gained by taking risks. The maximin principle finally clearly shows a precautionary approach based on ambiguity aversion and risk aversion: a high desire for security and heavy weight on worst outcomes and a low desire for potential and low weight on the best outcomes (Lopes, 1987). In fact, Rawls believes any “normal” and “reasonable” person would show a high level of risk aversion (1971: 144). See for other liberal‐egalitarian authors arguing for precaution in the face of risk: Armstrong (2013), and Shue (2014), Caney (2009), Gardiner (2006).

### 4.2 Optimism, risk‐seeking, and libertarianism

What would an optimistic and risk‐seeking person with a high desire for potential choose behind the veil of ignorance? Not only will an optimist believe the chances of turning up in the worst position to be very low, but she will also attach little credence to static conceptions of “bad” and “good” positions in society. We can always make the best of our circumstances; there are always opportunities for improvement. The true optimist is not an optimist because she believes that she herself possesses unique qualities by which she can change any situation to her advantage but is an optimist because she believes the world is benevolent. The glass is half‐full for everyone. In contrast to the precautionary approach taken by people with a more pessimistic, risk‐averse bias, people with a more optimistic, and risk‐seeking bias will apply trial‐and‐error. Not only does the optimist believe the world to be benevolent, but the optimist generally also believes in being in control of their life, that is, that their lives are determined by their personal decisions and efforts instead of external circumstances. This personality trait is called a high internal locus of control (Budria *et al.*, 2012; Rotter, 1954 and 1966).

Behind the veil of ignorance, such a person will therefore focus on negative rights and liberties, that is, on being protected from and being unrestricted by others in giving shape to one's own life, and be less interested in positive rights and liberties, that is, rights to help by others. This person would therefore agree to Rawls’ first principle of justice that “each person is to have an equal right to the most extensive basic liberty compatible with a similar liberty for others” (Rawls, 1971: 60) but would reject his difference principle, as it interferes with the first principle (Lomasky, 2005: 190; Tomasi, 2012). Therefore, the optimist feels much less need for collectively arranged social security: As long as you are not restrained by others, open doors will be found. According to Tomasi (2012), there is, moreover, more value in outcomes that are brought about by individuals’ own agency. Finally, optimists will have higher expectations of the benefits of capitalism and economic growth for the whole of society. This is not to say people may not be unrealistically optimistic (see Taylor & Brown, 1988; Weinstein, 1980 on optimism biases). In other words, the risk‐seeking person would arrive at the political philosophy of classical liberalism or libertarianism (see, e.g., Narveson, 1988; Nozick, 1974; Tomasi, 2012).

Few libertarians have explicitly justified their preferred organization of society by applying the thought‐experiment of the veil of ignorance (see, e.g., Lomasky, 2005). Drawing inspiration from libertarian authors such as Hayek (1960, 1976) and Rand (1971), however, several publications by Wildavsky illustrate how cognitive biases of optimism and being risk seeking lead to libertarianism.^11^

[T]rial‐and‐error risk taking, rather than risk aversion, is the preferable strategy for securing safety. Encouraging trial and error promotes resilience – learning from adversity how to do better – while avoiding restrictions that encourage the continuation of existing hazards. (Wildavsky, 1988: 2)

[I]t is not surprising that systems such as capitalism, based on incessant and decentralized trial and error, accumulate the most resources. Strong evidence from around the world demonstrates that such societies are richer and produce healthier people and a more vibrant natural environment. (Wildavsky & Wildavsky, 2008)

There are forms of government and ways of life that make it their explicit purpose to guard individuals against misfortune. The “social insurance state” and the “welfare state” are but a few of the prominent designations of regimes that attempt to guard their members against misfortune. Libertarianism, I take it, is not one of them. So long as individuals are able to try again, and there are no legal limits on transactions, failure serves a positive purpose. It cleanses the system. Without failure on the part of some participants there can be no success, no allocation of resources to higher order uses, no consumer sovereignty. For if there can be no individual failure, there can be no switching of resources away from activities that are unprofitable. Failure is more important then [sic] success in that it leaves the way open to try new and better combinations that meet more widespread preferences. (Wildavsky, 1982: 305).

### 4.3 Overconfidence in experts and optimization, and utilitarianism

The work by John Harsanyi (1953, 1975) offers a clear example how the cognitive biases of overconfidence in the feasibility of optimization results in utilitarian principles of justice. Harsanyi (1975: 595) argues against Rawls’ precautionary maximin strategy: “it is extremely irrational to make your behavior wholly dependent on some highly unlikely unfavorable contingencies *regardless of how little probability you are willing to assign to them*.” Instead, Harsanyi believes that behind Rawls’ veil of ignorance rationality would require the application of the expected‐utility maximization principle of Bayesian theory (Savage, 1954). The combination of expected‐utility maximation and the moral requirement of impartiality (as expressed by the thought experiment of the veil of ignorance) leads to utilitarianism, the moral and political principle that society should be organized such that the utility of all concerned is maximized. In such a society, maximum collective—and thus at the same time average—wellbeing is more important than liberal or libertarian individual rights. The interests or preferences of individuals might be sacrificed if this is to the benefit of the collective.

As mentioned earlier, Savage (1954) believed optimization on the basis of Bayesian decision theory was only possible in “small worlds” where all alternatives, consequences, and probabilities are known with certainty. This is not the case behind the veil of ignorance. Harsanyi believes, however, that we can always optimize on the basis of subjective probabilities, although these will inevitably reflect personal cognitive biases (Tversky & Kahneman, 1974). Moreover, Harsanyi assumes equal probabilities in the case of ambiguity in line with the so‐called principle of insufficient reason. According to this principle, first formulated by Jacob Bernoulli (1713), probabilities that are really unknown should be regarded as equal. Various authors have pointed out, however, that if there is no ground for assuming unequal probabilities there is as little rational ground to assume equal probabilities (see, e.g., Shrader‐Frechette, 1991).

From its first formulation by Jeremy Bentham (1781), utilitarians have expressed (over)confidence in the practical possibility to perform the utilitarian calculus (see, e.g., Hare, 1981; Mill, 1863; Singer, 1980). Determination of the right act after all requires a calculation on the basis of an inventory of all alternative actions and an inventory of all consequences of each individual act in terms of utility, happiness, or wellbeing for each affected individual. It requires not only knowledge of all consequences but also their probability. Utilitarianism thus puts a considerable trust in the ability of experts to acquire and deal with the enormous amounts of information required to perform the optimization process. It implies trust in social cost‐benefit analysis of policy measures and other evidence‐based analysis. Many authors have therefore criticized utilitarianism for its overconfidence that such optimization calculations could be performed in practice (see, e.g., Moore, 1903; Nagel, 1979; Williams, 1973; Elster & Roemer, 1991).

In response to the practical impossibility of performing the utilitarian calculus, various authors have proposed so‐called rule utilitarianism. Rule utilitarianism is the theory that a morally right action is simply an action conforming to that particular behavioral rule that would yield the highest expected social utility if it were followed by all morally motivated people in all similar situations (Harsanyi, 1985). Rule‐utilitarians might advocate basic rights on the basis of their social utility. Rule utilitarianism may therefore resemble both libertarianism as argued for by Narveson (1988) and liberalism as argued for by John Stuart Mill (1859).

## 5 CONCLUSION AND DISCUSSION

This article has aimed to make plausible that the same cognitive biases toward ambiguous risk guide both beliefs regarding nature's resilience and political preferences regarding the organization of society, thereby contributing to the explanation of their correlation in CT. The same pessimism and ambiguity aversion, for example, that make people doubt nature's resilience make them fight social inequality out of a sense of justice that they could have been on the losing side as well. The same overconfidence that we can defy ambiguity makes people believe that experts can determine “planetary boundaries” and that we can optimize our chances under utilitarian principles. Although CT does not explicitly link specific normative political theories to the myths of human nature or “ways of life,” there is a clear logical fit between CT's “egalitarian” and liberal egalitarianism, CT's “individualist” and libertarianism, CT's “hierarchist” and utilitarianism, and CT's fatalist and moral cynicism. The argument has been explorative, however, and further empirical and theoretical research is required.

The purpose of this article has not been to replace CT's explanation for the correlation between the archetypes. The existence of confirmation bias cannot be denied (Kahan, 2012, 2013; Klayman, 1995), that is, the tendency to search for, interpret, and favor information in a way that confirms or supports one's prior beliefs or values, including political preferences. Nevertheless, the analysis presented in this article gives reason to expect individualists to see fewer dangers than egalitarians, whereas CT so far has only expected individualists and egalitarians to see different dangers. Empirical research should provide clarity here, research in which people are directly tested for their cognitive biases toward ambiguous risk, such as optimism and pessimism, in relation to myths of nature and human nature.

The idea that cognitive biases toward ambiguous risks inevitably influence our moral/political intuitions has consequences for both normative and descriptive political philosophy. First of all, our moral intuitions about fairness are subconsciously determined by our tendency to put ourselves in other people's shoes. This partly explains why cognitive biases toward ambiguous risk determine our political preferences. Moreover, from the point of view of normative political philosophy, however, our cognitive biases are important. According to the method of reflective equilibrium, there is no inherent truth in a theory of justice independent from consistency with our considered moral judgments or intuitions in specific cases. But it appears that our cognitive biases toward ambiguous risk influence our moral judgments and intuitions, as exemplified and aroused by Rawls’ thought experiment of the veil of ignorance. If our moral intuitions are indeed influenced by cognitive biases, does this make our intuitions less reliable and the method of reflective equilibrium less applicable? These issues require further debate.

A final conclusion is that the myth of nature as perverse/tolerant appears to be linked to the bias of overconfidence and should be interpreted as the belief that experts can quantify an optimal approach in the epistemic mist. This belief therefore does not only encompass the belief in a safe zone or “planetary boundaries”—the common view in CT—but also the belief that economists can optimize our approach on the basis of cost‐benefit analysis. This may come as no surprise as economic analysis is strongly linked to the moral‐political view of utilitarianism. In the literature, an overconfidence bias has primarily been linked to overconfidence in one's own assessment in particular. Further research is therefore required into the nature of the cognitive biases that underlie overconfidence in experts being able to defy ambiguity. Certainly, from the point of policy makers, an eagerness to embrace the concept of planetary boundaries is understandable as it simplifies decision‐making and communication. How can this cognitive bias, which in effect denies ambiguity, be described, modeled, and measured?

## Footnotes

1 Note that the biases central in this article are general psychological biases toward risk, such as optimism and risk aversion, and should not be confused with the “cultural biases” described in CT. According to Swedlow et al. (2020: 2375), “Cultural biases consist of values and beliefs, including ideas and myths of nature, myths of human nature, ways of economizing, and preferences for the relational dimensions and patterns as well as for particular constructs of blame, envy, economic growth, scarcity, risk, and apathy.”

2 The myths of nature as fragile or benign strongly fit Mann's metaphors of the “wizard” and the “prophet” (Mann, 2018).

3 See Grendstad and Selle (2000) for doubts about this correlation.

4 See Flyvbjerg and Bester (2021) on overconfidence in cost‐benefit analysis.

5 The claim is not that tipping points do not exist, but that uncertainty about where they occur makes the relationship between human activities and resulting risks a continuous function again, thereby undercutting the idea of a “safe zone.” This blurs the distinction with the myth of nature as fragile.

6 See Wildavsky (1989), Lockhart and Wildavsky (1993), Wildavsky (1998) and Swedlow (2011) for efforts to apply CT to normative political philosophy.

7 Contractarians, such as Hobbes (1651) and Gaulthier (1987), on the other hand, believe it is fair to maximise one's own interests in a bargain with others on the basis of personal advantages.

8 In the case of *certain* risk, however, various authors have written on the influence of risk aversion on views on distributive justice (see, e.g., Kroll and Davidovitz, 2003; Carlsson et al., 2005; Ferrer‐i‐Carbonell et al., 2010; Chambers, 2012; Van Koten et al., 2013; Kameda et al., 2016; Kappes et al., 2016; Gaitán et al., 2024).

9 Please note that cognitive biases towards ambiguous risk do not make our moral intuitions unreliable in contrast to framing effects. The latter kind of cognitive biases give some philosophers, such as Sinnott‐Armstrong (2006), reason to reject the relevance of moral intuitions and thus the method of reflective equilibrium as well (see, e.g., Paulo, 2020).

10 On the basis of pessimism, ambiguity aversion, and risk aversion, we may also arrive at Dworkin's liberal‐egalitarianism (Dworkin, 1981). In contrast to Rawls, Dworkin proposes an insurance scheme to compensate people for unequal natural talents and impairments.

11 There is some irony that while CT explicitly claims that comparisons of risk filters lie beyond rational discussion, one of its founding fathers claims that the libertarian position based upon trial and error is superior to the liberal‐egalitarian position based upon precaution.

## References

[risa17668-bib-0001] Adams, J. (1995). Risk. UCL Press.

[risa17668-bib-0002] Angner, E. (2004). Revisiting Rawls: A theory of justice in the light of Levi's theory of decision. Theoria, 70(1), 3–21.

[risa17668-bib-0003] Armstrong, C. (2013). Natural resources: the demands of equality. Journal of Social Philosophy, 44(4), 331–347.

[risa17668-bib-0004] Arrow, K. J. (1963). The portfolio approach to the demand for money and other assets. The Review of Economics and Statistics, 45(1), Supplement, 24–31.

[risa17668-bib-0005] Beitz, C. R. (1975). Justice and international relations. Philosophy & Public Affairs, 4(4), 360–389.

[risa17668-bib-0006] Bentham, J. (1781). An introduction to the principles of morals and legislation. T. Payne and Son.

[risa17668-bib-0007] Bernoulli, D. (1738). Exposition of a new theory on the measurement of risk (Reprinted (1967)). Gregg Press.

[risa17668-bib-0008] Bernoulli, J. (1713). Ars conjectandi, opus posthumum: accedit tractatus de seriebus infinitis, et epistola Gallice scripta de ludo pilæ reticularis. Impensis Thurnisiorum Fratrum.

[risa17668-bib-0009] Biermann, F. (2007). ‘Earth system governance’ as a crosscutting theme of global change research. Global Environmental Change, 17(3–4), 326–337.

[risa17668-bib-0010] Biermann, F. , & Kim, R. E. (2020). The boundaries of the planetary boundary framework: a critical appraisal of approaches to define a “safe operating space” for humanity. Annual Review of Environment and Resources, 45(1), 497–521.

[risa17668-bib-0011] Boholm, Å. (1996). Risk perception and social anthropology: critique of cultural theory. Ethnos, 61(2), 159–178.

[risa17668-bib-0012] Breakwell, G. M. (2014). The psychology of risk. Cambridge University Press.

[risa17668-bib-0013] Buchak, L. (2017). Taking risks behind the veil of ignorance. Ethics, 127(3), 610–644.

[risa17668-bib-0014] Budria, S. , Ferrer‐i‐Carbonell, A. , & Ramos, X. (2012). Personality and dislike for inequality: Typifying inequality aversion with respect to locus of control. Mimeo.

[risa17668-bib-0015] Buttel, F. M. , & Flinn, W. L. (1978). The politics of environmental concern: The impacts of party identification and political ideology on environmental attitudes. Environment and Behavior, 10(1), 17–36.

[risa17668-bib-0016] Caney, S. (2009). Climate change and the future: Discounting for time, wealth, and risk. Journal of Social Philosophy, 40(2), 163–186.

[risa17668-bib-0017] Caney, S. (2010). Climate change and the duties of the advantaged. Critical Review of International Social and Political Philosophy, 13(1), 203–228. 10.1080/13698230903326331

[risa17668-bib-0018] Carlsson, F. , Daruvala, D. , & Johansson‐Stenman, O. (2005). Are people inequality—Averse, or just risk‐averse? Economica, 72(287), 375–396.

[risa17668-bib-0019] Chambers, C. (2012). Inequality aversion and risk aversion. Journal of Economic Theory, 147(4), 1642–1651.

[risa17668-bib-0020] Coad, A. , Nightingale, P. , Stilgoe, J. , & Vezzani, A. (2021). Editorial: the dark side of innovation. Industry and Innovation, 28(1), 102–112.

[risa17668-bib-0021] Cruz, S. M. (2017). The relationships of political ideology and party affiliation with environmental concern: A meta‐analysis. Journal of Environmental Psychology, 53, 81–91.

[risa17668-bib-0022] Dake, K. (1991). Orienting dispositions in the perception of risk: An analysis of contemporary worldviews and cultural biases. Journal of Cross‐Cultural Psychology, 22(1), 61.

[risa17668-bib-0023] Dake, K. (1992). Myths of nature: Culture and the social construction of risk. Journal of Social Issues, 48(4), 21–37.

[risa17668-bib-0024] De Coninck, T. , & Van De Putte, F. (2023). Original position arguments and social choice under ignorance. Theory and Decision, 94(2), 275–298.

[risa17668-bib-0025] Dimitrijevska‐Markoski, T. , & Nukpezah, J. A. (2023). COVID‐19 risk perception and support for COVID‐19 mitigation measures among local government officials in the US: A test of a cultural theory of risk. Administration & Society, 55(3), 351–380. 38603325 10.1177/00953997221147243 PMC9902793

[risa17668-bib-0026] Douglas, M. (1978). Cultural bias. Royal Anthropological Institute of Great Britain and Ireland.

[risa17668-bib-0027] Douglas, M. (1982). Cultural bias. In M Douglas (Ed.), In the active voice (pp. 183–254). Routledge and Kegan Paul.

[risa17668-bib-0028] Douglas, M. (1985). Risk acceptability according to the social sciences. Russell Sage Foundation.

[risa17668-bib-0029] Douglas, M. , & Wildavsky, A. B. (1982). Risk and culture. An essay on the selection of technical and environmental dangers. University of California Press.

[risa17668-bib-0030] Dworkin, R. (1981). What is equality? Part1: Equality of welfare. Philosophy and Public Affairs, 10(3), 185–246.

[risa17668-bib-0031] Ehrlich, P. R. (1968). The population bomb. Ballantine Books.

[risa17668-bib-0032] Ellis, R. J. , & Thompson, F. (1997). Culture and the environment in the Pacific Northwest. American Political Science Review, 91(4), 885–897.

[risa17668-bib-0033] Ellsberg, D. (1961). Risk, ambiguity, and the savage axioms. Quarterly Journal of Economics, 75, 643–669.

[risa17668-bib-0034] Elster, J. , & J. E. Roemer (Eds.). (1991). Interpersonal comparisons of well‐being. Cambridge University Press.

[risa17668-bib-0035] Evenson, R. E. , & Gollin, D. (2003). Assessing the impact of the Green Revolution, 1960 to 2000. Science, 300(5620), 758–762. 12730592 10.1126/science.1078710

[risa17668-bib-0036] Ferrer‐i‐Carbonell, A. , & Ramos, X. (2010), Inequality aversion and risk attitudes. In SOEP papers on multidisciplinary panel data research (Vol. 271) DIW.

[risa17668-bib-0037] Fischhoff, B. , Slovic, P. , Lichtenstein, S. , Read, S. , & Combs, B. (1978). How safe is safe enough? A psychometric study of attitudes towards technological risks and benefits. Policy Sciences, 9, 127–152.

[risa17668-bib-0038] Flyvbjerg, B. , & Bester, D. W. (2021). The cost‐benefit fallacy: Why cost–benefit analysis is broken and how to fix it. Journal of Benefit‐Cost Analysis, 12(3), 395–419.

[risa17668-bib-0039] Gaitán, A. , Aguiar, F. , & Viciana, H. (2024). The experimental turn in moral and political philosophy. In: Viciana, H. , Gaitán, A. , & Aguiar, F. (Eds.), Experiments in moral and political philosophy (pp. 1–19). Routledge.

[risa17668-bib-0040] Gardiner, S. M. (2006). A core precautionary principle. Journal of Political Philosophy, 14(1), 33–60.

[risa17668-bib-0041] Gardiner, S. M. (2011). Rawls and climate change: Does Rawlsian political philosophy pass the global test? Critical Review of International Social and Political Philosophy, 14, 125–151.

[risa17668-bib-0042] Gaus, G. , & Thrasher, J. (2015). Rational choice and the original position: The (many) models of Rawls and Harsanyi. In: T. Hinton (Ed.), The original position (pp. 39–58). Cambridge University Press.

[risa17668-bib-0043] Gauthier, D. (1987). Morals by agreement. Clarendon Press.

[risa17668-bib-0044] Gigerenzer, G. (2010). Moral satisficing: Rethinking moral behavior as bounded rationality. Topics in Cognitive Science, 2(3), 528–554. 25163875 10.1111/j.1756-8765.2010.01094.x

[risa17668-bib-0045] Grendstad, G. , & Selle, P. (2000). Cultural myths of human and physical nature: Integrated or separated? Risk Analysis, 20(1), 27–40. 10795336 10.1111/0272-4332.00003

[risa17668-bib-0046] Gustafsson, J. E. (2018). The difference principle would not be chosen behind the veil of ignorance. The Journal of Philosophy, 115(11), 588–604.

[risa17668-bib-0047] Hare, R. M. (1981). Moral thinking: Its levels, method, and point. Oxford University Press.

[risa17668-bib-0048] Harsanyi, J. C. (1953). Cardinal utility in welfare economics and in the theory of risk‐taking. Journal of Political Economy, 61(5), 434–435.

[risa17668-bib-0049] Harsanyi, J. (1975). Can the maximin principle serve as the basis for morality? A critique of John Rawls's theory. American Political Science Review, 69, 594–606.

[risa17668-bib-0050] Harsanyi, J. C. (1985). Rule utilitarianism, equality, and justice. Social Philosophy and Policy, 2(2), 115–127.

[risa17668-bib-0051] Hartmann, T. (2012). Wicked problems and clumsy solutions: Planning as expectation management. Planning Theory, 11(3), 242–256. 10.1177/1473095212440427

[risa17668-bib-0052] Hayek, F. A. (1944). The road to serfdom. Routledge.

[risa17668-bib-0053] Hayek, F. A. (1960). The constitution of liberty. University of Chicago Press.

[risa17668-bib-0054] Hayek, F. A. (1976). The mirage of social justice. In: Law, legislation, and liberty (Vol. 2, pp. 133–134). London: Routledge & Kegan Paul.

[risa17668-bib-0055] Hobbes, T. (1651). Leviathan. Printed for Andrew Crooke at the Green Dragon in St Pauls Church Yard.

[risa17668-bib-0056] Holling, C. S. (1978). Myths of ecological stability: Resilience and the Problem of Failure. In C. F. Smart & W. T. Stanbury (Eds.), Studies on crisis management (pp. 93–106). Butterworth & Co. Ltd.

[risa17668-bib-0057] Holling, C. S. (1986). The resilience of terrestrial ecosystems: local surprise and global change. In: W. C. Clark & R. E. Munn (Eds.), Sustainable development of the biosphere (pp. 292–317). Cambridge University Press.

[risa17668-bib-0058] Hume, D. (1740). In: L. A. Selby‐Bigge & P. H. Nidditch (Eds.), A treatise of human nature (2nd ed.) Oxford University Press.

[risa17668-bib-0059] IPCC . (2021). The physical science basis. Summary for policymakers. Intergovernmental Panel on Climate Change.

[risa17668-bib-0060] Johnson, B. B. , & Swedlow, B. (2020). Comparing cultural theory and cultural cognition theory survey measures to each other and as explanations for judged risk. Journal of Risk Research, 23(10), 1278–1300.

[risa17668-bib-0061] Johnson, B. B. , & Swedlow, B. (2021). Cultural theory's contributions to risk analysis: A thematic review with directions and resources for further research. Risk Analysis, 41(3), 429–455. 30970166 10.1111/risa.13299

[risa17668-bib-0062] Kahan, D. M. (2012). Cultural cognition as a conception of the cultural theory of risk. In S. Roeser , R. Hillerbrand , P. Sandin , & M. Petersen (Eds.), Handbook of risk theory: Epistemology, decision theory, ethics, and social implications of risk (pp. 725–759). Springer.

[risa17668-bib-0063] Kahan, D. M. , & Braman, D. (2006). Cultural cognition and public policy. Yale Law & Policy Review, 24, 149–157.

[risa17668-bib-0064] Kahan, D. M. , Slovic, P. , Braman, D. , & Gastil, J. (2006). Fear of democracy: A cultural critique of sunstein on risk. Harvard Law Review, 119, 1071–1109.

[risa17668-bib-0065] Kahan, D. M. (2013). Ideology, motivated reasoning, and cognitive reflection. Judgment and Decision Making, 8(4), 407–424.

[risa17668-bib-0066] Kahneman, D. , Slovic, P. , & Tversky, A. Eds. (1982). Judgment under uncertainty: Heuristics and biases. Cambridge university press. 10.1126/science.185.4157.1124 17835457

[risa17668-bib-0067] Kameda, T. , Inukai, K. , Higuchi, S. , Ogawa, A. , Kim, H. , Matsuda, T. , & Sakagami, M. (2016). Rawlsian maximin rule operates as a common cognitive anchor in distributive justice and risky decisions. Proceedings of the National Academy of Sciences, 113(42), 11817–11822. 10.1073/pnas.1602641113 PMC5081577 27688764

[risa17668-bib-0068] Kant, I. (1785). Groundwork of the metaphysics of morals, Translated by H. J. Paton . Harper and Row.

[risa17668-bib-0069] Kappes, A. , Kahane, G. , & Crockett, M. J. (2016). From risk to fairness. Proceedings of the National Academy of Sciences, 113(42), 11651–11653. 10.1073/pnas.1614111113 PMC5081645 27791048

[risa17668-bib-0070] Klayman, J. (1995). Varieties of confirmation bias. Psychology of Learning and Motivation, 32, 385–418.

[risa17668-bib-0071] Knight, C. (2023). Reflective equilibrium. In E. N. Zalta & U. Nodelman (Eds.), The Stanford encyclopedia of philosophy (Winter 2023 Edition). Stanford. https://plato.stanford.edu/archives/win2023/entries/reflective‐equilibrium/

[risa17668-bib-0072] Knutti, R. , Rogelj, J. , Sedláček, J. , & Fischer, E. M. (2016). A scientific critique of the two‐degree climate change target. Nature Geoscience, 9(1), 13–18.

[risa17668-bib-0073] Kroll, Y. , & Davidovitz, L. (2003). Inequality aversion versus risk aversion. Economica, 70(277), 19–29.

[risa17668-bib-0074] Kymlicka, W. (2002). Contemporary political philosophy: An introduction. Oxford University Press.

[risa17668-bib-0075] Lambert, P. , & Weale, A. (1981). Equality, risk‐aversion and contractarian social choice. Theory and Decision, 13, 109–127.

[risa17668-bib-0076] Langer, E. J. (1975). The illusion of control. Journal of Personality and Social Psychology, 32(2), 311.

[risa17668-bib-0077] Langford, I. H. , Georgiou, S. , Bateman, I. J. , Day, R. J. , & Turner, R. K. (2000). Public perceptions of health risks from polluted coastal bathing waters: A mixed methodological analysis using cultural theory. Risk Analysis, 20(5), 691–704. 11110215 10.1111/0272-4332.205062

[risa17668-bib-0078] Levin, M. (1978). The Problem of knowledge in the original position. Auslegung: A Journal of Philosophy, 5(3), 147–160.

[risa17668-bib-0079] Linsley, P. M. , & Shrives, P. J. (2009). Mary Douglas, risk and accounting failures. Critical Perspectives on Accounting, 20(4), 492–508.

[risa17668-bib-0080] Lockhart, C. , & Wildavsky, A. (1993). The ‘multicultural’ Mill. Utilitas, 5(2), 255–273.

[risa17668-bib-0081] Lomasky, L. E. (2005). Libertarianism at twin Harvard. Social Philosophy and Policy, 22(1), 178–199.

[risa17668-bib-0082] Lomborg, B. (2003). The skeptical environmentalist: measuring the real state of the world (Vol. 1). Cambridge University Press.

[risa17668-bib-0083] Lopes, L. L. (1987). Between hope and fear: The psychology of risk. Advances in Experimental Social Psychology, 20(3), 255–295.

[risa17668-bib-0084] Malthus, T. (1798). In A. Flew (Ed.), An essay on the principle of population (1st ed.). Penguin English Library.

[risa17668-bib-0085] Mamadouh, V. (1999). Grid‐group cultural theory: An introduction. GeoJournal, 47, 395–409.

[risa17668-bib-0086] Mann, C. C. (2018). The wizard and the prophet: Two remarkable scientists and their dueling visions to shape tomorrow's world. Alfred A. Knopf.

[risa17668-bib-0087] Marris, C. , Langford, I. H. , & O'Riordan, T. (1998). A quantitative test of the cultural theory of risk perceptions: Comparison with the psychometric paradigm. Risk Analysis, 18(5), 635–647. 9853396 10.1023/b:rian.0000005937.60969.32

[risa17668-bib-0088] Meadowcroft, J. (2009). What about the politics? Sustainable development, transition management, and long term energy transitions. Policy Sciences, 42, 323–340.

[risa17668-bib-0089] Mill, J. S. (1859). On liberty. John W. Parker and Son.

[risa17668-bib-0090] Mill, J. S. (1863). Utilitarianism. John W. Parker and Son.

[risa17668-bib-0091] Moore, G. E. (1903). Principia ethica. Cambridge University Press.

[risa17668-bib-0092] Nagel, T. (1979). Mortal questions. Cambridge university press.

[risa17668-bib-0093] Narveson, J. (1988). The libertarian idea. Temple University Press.

[risa17668-bib-0094] Narveson, J. (1998). An interview with Jan Narveson. Cogito, 12(2), 93–102.

[risa17668-bib-0095] Nozick, R. (1974). Anarchy, state, and utopia. Basic Books.

[risa17668-bib-0096] Oltedal, S. , Moen, B. E. , Klempe, H. , & Rundmo, T. (2004). Explaining risk perception: An evaluation of cultural theory. Trondheim: Norwegian University of Science and Technology, 85, 1–33.

[risa17668-bib-0097] Opschoor, J. B. , 1987. Duurzaamheid en verandering (sustainability and change). Free University.

[risa17668-bib-0098] Oreskes, N. , & Conway, E. M. (2011). Merchants of doubt: How a handful of scientists obscured the truth on issues from tobacco smoke to global warming. Bloomsbury Publishing USA.

[risa17668-bib-0099] Oskamp, S. (1965). Overconfidence in case‐study judgements. Journal of Consulting Psychology, 29, 261–265. 14303514 10.1037/h0022125

[risa17668-bib-0100] Paulo, N. (2020). The unreliable intuitions objection against reflective equilibrium. The Journal of Ethics, 24(3), 333–353.

[risa17668-bib-0101] Peres, R. Á. (2021). The Harsanyi–Rawls debate: Political philosophy as decision theory under uncertainty. Manuscrito, 44, 89–127.

[risa17668-bib-0102] Peters, E. , & Slovic, P. (1996). The role of affect and worldviews as orienting dispositions in the perception and acceptance of nuclear power. Journal of Applied Social Psychology, 26(16), 1427–1453.

[risa17668-bib-0103] Pinker, S. (2018). Enlightenment now: The case for reason, science, humanism, and progress. Penguin.

[risa17668-bib-0104] Poortinga, W. , Steg, L. , & Vlek, C. (2002). Environmental risk concern and preferences for energy‐saving measures. Environment and Behavior, 34(4), 455–478.

[risa17668-bib-0105] Pratt, J. (1964). Risk aversion in the small and in the large. Econometrica, 32(1), 122–136.

[risa17668-bib-0106] Rand, A. (1971), The anti‐industrial revolution. In The new left: The Anti‐industrial revolution (pp. 127–151). New American Library.

[risa17668-bib-0107] Rawls, J. (1971). A theory of justice. Harvard University Press.

[risa17668-bib-0108] Ridley, M. 2010. The rational optimist. How prosperity evolves. HarperCollins Publishers.

[risa17668-bib-0109] Rockström, J. , Steffen, W. , Noone, K. , Persson, Å. , Chapin, F. S. , Lambin, E. F. , Lenton, T. M. , Scheffer, M. , Folke, C. , Schellnhuber, H. J. , Nykvist, B. , de Wit, C. A. , Hughes, T. , van der Leeuw, S. , Rodhe, H. , Sörlin, S. , Snyder, P. K. , Costanza, R. , Svedin, U. , … & Foley, J. A. (2009). A safe operating space for humanity. Nature, 461(7263), 472–475. 19779433 10.1038/461472a

[risa17668-bib-0110] Roser, M. , Ritchie, H. , & Rosado, P. (2023). “Food Supply” Published online at OurWorldinData.org. Retrieved from: https://ourworldindata.org/food-supply [Online Resource]

[risa17668-bib-0111] Rosling, H. (2019). Factfulness: Ten reasons we're wrong about the world—And why things are better than you think. Flammarion.

[risa17668-bib-0112] Rotter, J. B. (1954). Social learning and clinical psychology. Prentice‐Hall.

[risa17668-bib-0113] Rotter, J. B. (1966). Generalized expectancies for internal versus external control of reinforcement. Psychological Monographs: General and Applied, 80(1), 1–28. 5340840

[risa17668-bib-0114] Savage, L. J. (1954). The foundations of statistics. John Wiley and Sons.

[risa17668-bib-0115] Schwarz, M. , & Thompson, M. (1990). Divided we stand: Redefining politics, technology, and social choice. University of Pennsylvania Press.

[risa17668-bib-0116] Shrader‐Frechette, K. S. (1991). Risk and Rationality. Philosophical Foundations for Populist Reforms. University of California Press.

[risa17668-bib-0117] Shue, H. (1999). Global environment and international inequality. International Affairs, 75(3), 531–545.

[risa17668-bib-0118] Shue, H. (2014). Climate justice: Vulnerability and protection. Oxford University Press.

[risa17668-bib-0119] Siebert, H. (1982). Nature as a life support system. Renewable resources and environmental disruption. Zeitschrift Für Nationalökonomie/Journal of Economics, 42(2), 133–142.

[risa17668-bib-0120] Simon, J. L. (1996). The ultimate resource 2. Princeton University Press.

[risa17668-bib-0121] Singer, P. (1980). Practical ethics. Cambridge University Press. 39946524

[risa17668-bib-0122] Sinnott‐Armstrong, W. (2006). Moral intuitionism meets empirical psychology. In: T Horgan & M Timmons (Eds.), Metaethics after Moore (pp. 339–365). Oxford University Press. 10.1093/acprof:oso/9780199269914.003.0016

[risa17668-bib-0123] Sjöberg, L. (1996). A discussion of the limitations of the psychometric and cultural theory approaches to risk perception. Radiation Protection Dosimetry, 68(3–4), 219–225.

[risa17668-bib-0124] Sjöberg, L. (1997). Explaining risk perception: An empirical and quantitative evaluation of cultural theory. Risk Decision and Policy, 2(2), 113–130.

[risa17668-bib-0125] Sjöberg, L. (1998). World views, political attitudes, and risk perception. Risk: Health, Safety and Environment, 9, 137–152.

[risa17668-bib-0126] Sjöberg, L. (2002). Are received risk perception models alive and well? Risk Analysis, 22(4), 665–669. 12224740 10.1111/0272-4332.00058

[risa17668-bib-0127] Slovic, P. (1987). Perception of risk. Science, 236(4799), 280–285. 3563507 10.1126/science.3563507

[risa17668-bib-0128] Smith, A. (1759). The theory of moral sentiments. Oxford University Press.

[risa17668-bib-0129] Swedlow, B. (2011). A cultural theory of politics: editor's introduction: Cultural theory's contributions to political science. PS: Political Science & Politics, 44(4), 703–710.

[risa17668-bib-0130] Swedlow, B. , Ripberger, J. T. , Liu, L. Y. , Silva, C. L. , Jenkins‐Smith, H. , & Johnson, B. B. (2020). Construct validity of cultural theory survey measures. Social Science Quarterly, 101(6), 2332–2383.

[risa17668-bib-0131] Tansey, J. , & Rayner, S. (2009). Cultural theory and risk. In R. L. Heath & H. D. O'Hair (Eds.), Handbook of risk and crisis communication (pp. 53–79). Routledge.

[risa17668-bib-0132] Taylor, S. E. , & Brown, J. D. (1988). Illusion and well‐being: A social psychological perspective on mental health. Psychological Bull., 103, 193–210. 3283814

[risa17668-bib-0133] Tenner, E. (1997). Why things bite back: Technology and the revenge of unintended consequences. Vintage.

[risa17668-bib-0134] Thompson, M. , Ellis, R. , & Wildavsky, A. (1990). Cultural theory. Westview.

[risa17668-bib-0135] Tomasi, J. (2012). Free market fairness. Princeton University Press.

[risa17668-bib-0136] Trivers, R. L. (1971). The evolution of reciprocal altruism. The Quarterly Review of Biology, 46(1), 35–57.

[risa17668-bib-0137] Tversky, A. , & Kahneman, D. (1974). Judgment under uncertainty: Heuristics and biases. Science, 185(4157), 1124–1131. 17835457 10.1126/science.185.4157.1124

[risa17668-bib-0138] Van Koten, S. , Ortmann, A. , & Babicky, V. (2013). Fairness in risky environments: theory and evidence. Games, 4(2), 208–242.

[risa17668-bib-0139] Verweij, M. , & Thompson, M. (Eds.). (2006). Clumsy solutions for a complex world: Governance, politics, and plural perceptions. Palgrave Macmillan.

[risa17668-bib-0140] Vickrey, W. (1945). Measuring marginal utility by reactions to risk. Econometrica, 13(4), 319–333.

[risa17668-bib-0141] Weinstein, N. D. (1980). Unrealistic optimism about future life events. Journal of Personality and Social Psychology, 39(5), 806.

[risa17668-bib-0142] Wildavsky, A. (1982). Pollution as moral coercion: Culture, risk perception, and libertarian values. Cato Journal, 2(1), 305–326.

[risa17668-bib-0143] Wildavsky, A. B. (1988). Searching for safety (Vol. 10). Transaction publishers.

[risa17668-bib-0144] Wildavsky, A. (1989). If institutions have consequences, why don't we hear about them from moral philosophers? American Political Science Review, 83(4), 1343–1351.

[risa17668-bib-0145] Wildavsky, A. (1998). In S. K. Chai & B. Swedlow (Eds.), Culture and social theory. Transaction Publishers.

[risa17668-bib-0146] Wildavsky, A. , & Wildavsky, A. (2008). Risk and safety. In The concise encyclopedia of economics . http://www.econlib.org/library/Enc/RiskandSafety.html

[risa17668-bib-0147] Williams, B. (1973). A critique of utilitarianism. In J. J. C. Smart & B. Williams (Eds.), Utilitarianism: For and against. Cambridge University Press.

[risa17668-bib-0148] Wissenburg, M. (2019). The concept of nature in libertarianism. Ethics, Policy & Environment, 22(3), 287–302.

